# Time-restricted feeding improves blood glucose and insulin sensitivity in overweight patients with type 2 diabetes: a randomised controlled trial

**DOI:** 10.1186/s12986-021-00613-9

**Published:** 2021-10-07

**Authors:** Tingting Che, Cheng Yan, Dingyuan Tian, Xin Zhang, Xuejun Liu, Zhongming Wu

**Affiliations:** 1 NHC Key Laboratory of Hormones and Development, Tianjin Key Laboratory of Metabolic Diseases, Chu Hsien-I Memorial Hospital & Tianjin Institute of Endocrinology, Tianjin Medical University, Tianjin, 300134 China; 2 Department of Neurology, Chu Hsien-I Memorial Hospital, Tianjin Medical University, Tianjin, 300134 China

**Keywords:** Time-restricted feeding, Overweight, Weight loss, Type 2 diabetes

## Abstract

**Background:**

Time-restricted feeding is an emerging dietary intervention that is becoming increasingly popular. There are, however, no randomised clinical trials of time-restricted feeding in overweight patients with type 2 diabetes. Here, we explored the effects of time-restricted feeding on glycaemic regulation and weight changes in overweight patients with type 2 diabetes over 12 weeks.

**Methods:**

Overweight adults with type 2 diabetes (n = 120) were randomised 1:1 to two diet groups: time-restricted feeding (n = 60) or control (n = 60). Sixty patients participated in a 10-h restricted feeding treatment program (ad libitum feeding from 8:00 to 18:00 h; fasting between 18:00 and 8:00 h) for 12 weeks.

**Results:**

Haemoglobin A1c and body weight decreased in the time-restricted feeding group (− 1.54% ± 0.19 and − 2.98 ± 0.43 kg, respectively) relative to the control group over 12 weeks (*p* < 0.001). Homeostatic model assessment of β-cell function and insulin resistance changed in the time-restricted feeding group (0.73 ± 0.21, *p* = 0.005; − 0.51 ± 0.08, *p* = 0.02, respectively) compared with the control group. The medication effect score, SF-12 score, and the levels of triglycerides, total cholesterol and low-density lipoprotein cholesterol were improved in the time-restricted feeding group (− 0.66 ± 0.17, *p* = 0.006; 5.92 ± 1.38, *p* < 0.001; − 0.23 ± 0.08 mmol/L, *p* = 0.03; − 0.32 ± 0.07 mmol/L, *p* = 0.01; − 0.42 ± 0.13 mmol/L, *p* = 0.02, respectively) relative to the control group. High-density lipoprotein cholesterol was not significantly different between the two groups.

**Conclusion:**

These results suggest that 10-h restricted feeding improves blood glucose and insulin sensitivity, results in weight loss, reduces the necessary dosage of hypoglycaemic drugs and enhances quality of life. It can also offer cardiovascular benefits by reducing atherosclerotic lipid levels.

*Trial registration*: This study was registered with the Chinese Clinical Trial Registry (ChiCTR-IPR-15006371).

**Supplementary Information:**

The online version contains supplementary material available at 10.1186/s12986-021-00613-9.

## Introduction

Diabetes mellitus has become an important global public health issue; it imposes a huge economic burden on the global healthcare system of $827 billion a year. Approximately 90% of people with diabetes have type 2 diabetes, which is often associated with being overweight or obese. This crisis will continue until a solution is found [1, 2]. In fact, the early observation linking calorie restriction (CR) to improved health is now a century old. McCay found that pups fed a limited diet lived longer than pups fed randomly [3]. The positive effects of CR on longevity and health have been demonstrated in many model organisms, such as fruit flies, mice and primates [4–6]. In addition, CR interventions can prevent and treat a variety of metabolic disorders, including diabetes [7–9].

While CR has many benefits, this type of diet may be difficult because it requires a vigilant daily calorie count [10]. Intermittent fasting, as an alternative to calorie restriction, has become increasingly popular over the past few decades [11, 12]. Intermittent fasting is divided into three subtypes [13]: alternate-day fasting, the 5:2 diet, and time-restricted feeding (TRF). Alternate-day fasting is defined as alternating between "fasting days" and “free feast days". The 5:2 diet involves fasting for just two days a week, followed by five free eating days. These two diets require a strict calorie count on fasting days and a calorie limit of 800 kcal. Unlike these two diets, time-limited feeding (TRF) does not require individuals to deliberately count calories and monitor food intake. TRF only restricts eating time to 4–12 h per day, where one does not consume any calories during the remaining hours of the day [14]. Therefore, as an emerging dietary strategy, TRF has attracted great attention.

To date, more than a dozen animal studies have examined the effects of TRF on metabolic disease [15, 16]. Gill [17] subjected Drosophila melanogaster adults to 12-h TRF of a standard cornmeal diet for 5 weeks. Their endurance, motor control and cardiac function improved significantly. For cafeteria diet-induced obesity in rats, the 8-h TRF regimen for 16 weeks is an effective strategy to enhance body weight gain, lipid profiles, and atherogenic indices [18]. In mutant mice, 10-h TRF for 12 weeks prevented obesity and metabolic syndrome [19]. Mice fed 8-h TRF for 16 weeks were protected against obesity, hyperinsulinemia, hepatic steatosis, and inflammation, and their motor coordination was improved [20]. Thus, TRF has multiple metabolic benefits, preventing chronic disease in mice and flies and, more importantly, reversing the consequences of obesity [21] and aging [22].The beneficial effects were also evident in high-fat fed mice when TRF was administered 5 days a week and free access to food was allowed on weekends [23].

The effects of TRF in humans have been poorly studied. To date, TRF has been studied in only seven human trials [24–30]. Human data on the benefits of TRF have focused on healthy humans who are overweight or obese [24, 25] [28–30]. Studies in overweight people showed that 10–12-h TRF for 10 weeks decreased fat mass and fasting plasma glucose concentration [29], and 10-h TRF for 12 weeks reduced visceral fat [30]. However, few studies have been performed on people with metabolic disorders. A single-arm trial in people with metabolic syndrome supported that 10-h TRF for 12 weeks induced reductions in body weight, adiposity, lipaemia and blood pressure [27]. In men with prediabetes in a crossover study, 6-h TRF for 5 weeks reduced signs of IR [26].

However, TRF as a behavioural intervention has never been studied in patients with type 2 diabetes. It is not known whether patients who have already received pharmacotherapy can benefit from adopting TRF. Previous studies have shown that further narrowing of the feeding window brings no additional benefits [28]. Therefore, we designed a 12-week 10-TRF intervention in overweight patients with type 2 diabetes. We hypothesised that 10-h TRF would improve blood glucose levels. It is also not known whether the types and amounts of antidiabetic drugs could be reduced or even stopped after adopting TRF as a therapy.

Our research is the first randomised controlled clinical trial to explore the effect of TRF on type 2 diabetes. We hypothesised that compared with the control condition, a 10-h TRF intervention for 12 weeks would result in a greater improvement in glucose regulation and insulin sensitivity. We also anticipated weight loss and improvements in cardiovascular disease (CVD) risk markers, such as atherogenic lipid levels. Finally, we expected that the 10-h TRF group would have better quality of life and require fewer medications.

## Methods

### Patients

**Fig. 1:**
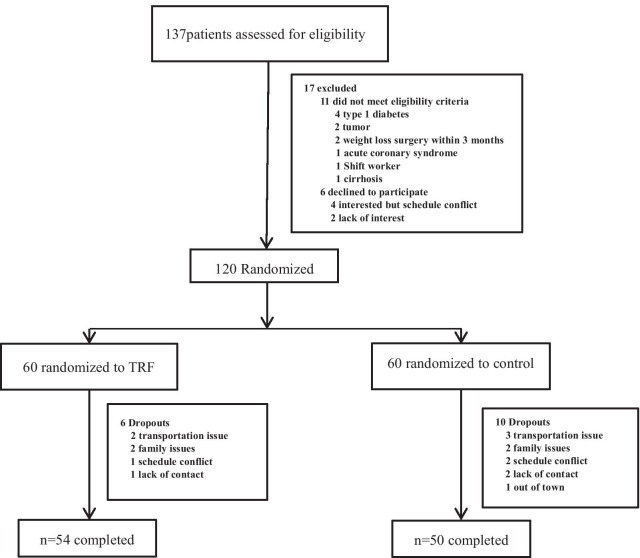
Flow diagram of the study participants from eligibility criteria screening to study completion. A total of 137 people were screened, of whom 17 were excluded based on the inclusion and exclusion criteria. A total of 120 participants were randomly assigned to a 10-h TRF or a control group. At the end of the trial, 54 participants in the 10-h TRF group had completed treatment, and 50 participants in the control group had completed treatment

Researchers recruited subjects from diabetes clinics by placing advertisements around the Zhu Xianyi Hospital of Tianjin Medical University. Participants were screened by questionnaire and body mass index (BMI) assessment. A total of 137 participants (Fig. 1) provided consent and were evaluated for eligibility. Of these, 17 were excluded according to the inclusion and exclusion criteria. The inclusion criteria were as follows: type 2 diabetes according to the WHO's 1999 Diabetes Diagnostic Criteria; BMI ≥ 25 kg/m^2^; age between 18 and 70 years; stable weight for 3 months prior to the beginning of the study (gain or loss < 2 kg); and ability to complete this study independently. The exclusion criteria were as follows: previous weight loss surgery; pregnancy or intent to become pregnant; moderate or severe chronic hepatorenal diseases or cardio-cerebrovascular diseases; current acute complications of diabetes, such as diabetic ketosis, hyperglycaemia and hypertonicity; in the past 3 months, stress diseases such as surgery, trauma, and cardiovascular and cerebrovascular events; and mental disorders requiring antipsychotic drugs.

The study started in APR 2019 and was completed by December 2019. The clinical trial number is ChiCTR-IPR-15006371. The experimental protocol was approved by the Ethics Committee of Tianjin Medical University, and all research participants provided written informed consent to participate in the trial. The study was conducted in accordance with the Helsinki Declaration of 1964 and its subsequent amendments.

### Randomisation and bias minimisation

Patients were assigned in a 1:1 ratio to the TRF therapy or control group. Assignment of treatment groups was based on a serially numbered label created using an electronic random number generator. The labels were placed in corresponding numbered opaque envelopes by the statistician who did not participate in the inclusion of patients or the subsequent experimental process. Investigators were masked to group allocation, but masking to the TRF intervention implementer was not possible. There was an independent assessment team, and none of the assessment staff were informed of the assignment of participants. Clinical tests were conducted in separate buildings, and participants were often reminded to not disclose their grouping to the assessors. Trial groups were masked for the analysis by an independent statistician, and those performing the analysis were unaware of the group allocation.

### Experimental design

**Fig. 2:**
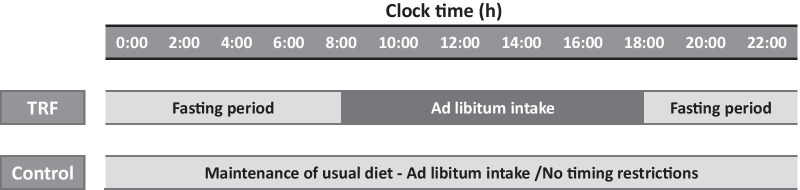
A time-limited feeding intervention was administered for 12 weeks. The 10-h TRF group was free to eat from 8:00 to 18:00 every day (fasting for 14 h). The control group was instructed to continue their usual eating pattern without any time restrictions on eating

A 14-week trial was conducted to compare the influences of 10-h TRF with those of the control condition. The trial consisted of a 2-week baseline weight stabilisation period and a 12-week TRF intervention period. The 10-h TRF group fed freely from 8:00 to 18:00 and fasted from 18:00 to 8:00 daily (a 14-h fast) (Fig. 2) in the 12-week intervention. TRF participants did not need to restrict caloric intake during the feeding window. In the fasting period, TRF participants are only allowed to intake water or tea without any calories. The control group was asked to maintain their normal diet throughout the trial. However, to prevent any investigator interaction bias, the frequency at which the control group visited the research centre was the same as that of the 10-h TRF group.

### Medication management

Drug management protocols were developed by endocrinologists. All participants were supervised by intervention implementers in consultation with endocrinologists. All participants were given capillary blood glucose metres. All participants were asked to measure their blood glucose levels at their fingers at their daily fasting and before going to bed. If the fasting or bedtime finger blood glucose reading was less than 8 mmol/L, the participant had their insulin reduced by approximately 4 units per day. The dose of insulin did not change if the blood glucose reading was higher than 8 mmol/L and less than 9 mmol/L. If the blood glucose level was higher than 9 mmol/L, the endocrinologist adjusted the participant’s medication. To reduce bias, endocrinologists did not know the group of the participant. Participants were asked to contact an implementer immediately if their blood sugar levels fell below 3.9 mmol/L. Implementers consulted with endocrinologists over the phone to make changes. Doses were recorded daily, and changes were quantified using the medication efficacy score (MES) [31]. The MES was calculated as (actual drug dose/maximum drug dose) × drug mean adjustment factor. A smaller MES corresponded to a reduced dose of diabetes medication. For example, an MES change of 0.5 is equivalent to a decrease of 1000 mg of metformin hydrochloride.

### Outcome measures

The primary outcomes were haemoglobin A1c (HbA1c), fasting plasma glucose and body weight. The secondary outcomes were homeostatic model assessment of β-cell function (HOMA-β), insulin resistance (HOMA-IR), MES, SF-12 score, adverse events and cardiovascular risk lipid markers (including triglycerides, total cholesterol, low-density lipoprotein (LDL) cholesterol and high-density lipoprotein (HDL) cholesterol.

#### Body weight and BMI

Body weight was assessed to the nearest 0.1 kg of participants without shoes and in light clothing using a digital scale (Shanghai Yaohua, XK3190-A12^+^E). Height was evaluated using a sitting height metre (Shanghai Keda, TZG) to the nearest 1 cm. BMI was calculated as weight (kg)/height (m)^2^.

#### Adherence to the TRF protocols

A daily log was used to measure adherence by having each participant record the time at which caloric intake was started and stopped every day. If the records showed that the participant ate only within the given 10 h, that day was marked as "consistent." If the records showed eating outside of the arranged 10-h feeding window, that day was marked as "non-consistent." The number of days per week was used to assess compliance with the TRF diet. Throughout the pilot period, TRF participants met weekly with the research supervisor. At each meeting, the research director reviewed their dietary adherence records, highlighted the importance of eating and addressed any problems they observed to improve compliance.

#### Blood analyses

All fasting blood analyses were conducted at baseline (week 1 of the study) and at 12 weeks (week 14 of the study). Fasting plasma glucose, insulin, HbA1c, triglycerides, total cholesterol, LDL cholesterol and HDL cholesterol concentrations were measured by the laboratory of Zhu Xianyi Hospital. β-cell function and IR were assessed using the homeostasis model assessment (HOMA) method by applying the following formulas: [HOMA-β = 20 × fasting insulin (mlU/ml)/(fasting glucose -3.5)] and [HOMA-IR = fasting insulin (mlU/ml) × fasting glucose (mmol/L)/22.5].

#### Sleep analysis

Sleep data were collected by means of a self-administered questionnaire.

#### Dietary intake and physical activity

A seven-day diet record was completed at baseline and the end of the trial. A nutritionist provided guidance on how to estimate portion sizes and keep detailed food records to obtain accurate dietary intake. Participants were asked to calculate the amount of food consumed using household measurement tools (i.e., a cup or spoon). The time of eating was also logged in the food record. Another nutritionist calculated the total for the day. The physical activity level of all participants was asked to be maintained throughout the trial. To avoid hypoglycaemia, participants in the TRF group were asked to exercise outside of the fasting window. Steps were measured using a belt-mounted pedometer, which participants had to wear at all times.

## Statistical analysis

We hypothesised that the 10-h TRF group would drop 5% of their body weight and that the control group would lose 2% after 12 weeks to calculate the sample size [24]. If 90% power was anticipated to discover a distinct superiority of 3% of body weight for the 10-h TRF group patients, n = 23 participants per group was calculated. We estimated a dropout rate of 20%. Thus, we assumed that 58 patients (n = 29 each group) would finish the clinical trial. However, we recruited 120 participants (n = 60 per group), which was a relatively large sample.

Outcome analyses used the intention-to-treat principle and involved all participants in the group to which they were randomised. The normality tests were conducted in the model, and nonnormally distributed data were converted into a normal distribution by natural logarithm and then analysed statistically. Independent samples t tests and Pearson χ^2^ tests were used to analyse differences between groups at baseline. To determine the effects of time-restricted feeding, we used repeated measures analysis of variance. Pearson correlation was used to determine independent factors associated with major outcome measures. Analyses were performed using SPSS, version 25 (IBM Corp., Armonk, NY, USA), and the data are shown as the mean (standard error of the mean). A 2-tailed *p* < 0.05 was considered significant.

## Results

### Participants

As shown in Fig. 2, 137 participants were recruited to the study. Of those, 11 were excluded based on the inclusion and exclusion criteria. Four patients were interested, but their schedules conflicted, and two patients lost interest later. The specific items for the inclusion or exclusion criteria are described in the Methods section. A total of 120 patients were randomised into the 10-h TRF group (n = 60) or the control group (n = 60). A total of 54 participants in the 10-h TRF group and 50 in the control group completed the study. No one withdrew from the study because they did not like the TRF intervention (Fig. 1). Table 1 indicates the baseline characteristics of all participants and completers. At baseline, in all participant and completion analyses, there was no significant difference between the 10-h TRF group and the control group in terms of primary outcome measures or any secondary outcome measure. There were also no significant differences in sleep duration or physical activity, which may lead to bias.

**Table 1:** General characteristics of the study participants

	TRF group (n = 60)	Control group (n = 60)	*p*^a^
Age (y)	48.21 ± 9.32	48.78 ± 9.56	0.25
Female	29 (48.33%)	26 (43.33%)	0.58^b^
Male	31 (51.67%)	34 (56.67%)	
Duration of diabetes (y)	4.86 ± 1.27	5.06 ± 1.46	0.15
*Diabetes medications (%)*			
Diet	20 (33.33%)	18 (30.00%)	0.69^b^
OHA	42 (70.00%)	46 (76.67%)	
Insulin	19 (31.67%)	15 (25.00%)	
Activity count (steps/d)	6457.43 ± 231.20	6405.00 ± 122.68	0.13
Sleep duration (h)	7.57 ± 0.50	7.55 ± 0.51	0.72

Data are means ± SDs

OHA, oral hypoglycaemic agents

^a^Obtained from the independent t test

^b^Obtained from the chi-square test

### Adherence to eating pattern and adverse effects

At baseline, the average eating window was not notably different (*p* = 0.62) between the 10-h TRF (15.02 ± 1.23 h) and control (15.24 ± 1.41 h) groups. The eating window was significantly reduced by 29.49% (4.43 ± 1.16 h) compared with the baseline value (*p* < 0.001). Compliance with the TRF intervention was excellent (Additional file 1: Figure S1). TRF participants adhered to the intervention by delaying the time of their first caloric intake and consuming their last calories at an earlier time in the day. The first caloric intake was delayed by 2.01 ± 1.18 h, and their last caloric intake was advanced by 2.51 ± 1.27 h relative to baseline. Mean daily caloric intake, which was self-reported and estimated, decreased △ = − 531 ± 102 kcal/d [28%] in the 10-h TRF group compared with the control group △ = − 76 ± 42 kcal/d [5%], *p* < 0.05 (Table 2). Regarding adverse effects, participants in the 10-h TRF intervention group did not experience any adverse events, including headaches, thirst, and diarrhoea. The number of hypoglycaemic events was one in the control group; there were no hypoglycaemic events in the TRF group.

**Table 2:** Dietary intake at study baseline and after the 12-week intervention

Variable	TRF group			Control group		
Dietary intake	Baseline	Week 12	Change	Baseline	Week 12	Change
Energy (kcal)	1876 ± 202	1345 ± 120	− 531 ± 102*	1672 ± 117	1596 ± 105	− 76 ± 42
Protein (%)	18 ± 1	19 ± 1	1 ± 1	19 ± 1	18 ± 1	− 1 ± 1
Carbohydrates (%)	52 ± 1	49 ± 2	− 3 ± 2	51 ± 1	52 ± 3	1 ± 1
Total sugar (%)	12 ± 1	9 ± 1	− 3 ± 1	11 ± 1	12 ± 1	1 ± 1
Fat (%)	30 ± 2	32 ± 3	2 ± 2	30 ± 1	30 ± 1	0 ± 1
Saturated fat (%)	11 ± 1	12 ± 1	1 ± 1	12 ± 2	11 ± 1	− 1 ± 1
Monounsaturated fat (%)	10 ± 1	9 ± 1	− 1 ± 1	11 ± 2	10 ± 3	− 1 ± 2
Polyunsaturated fat (%)	9 ± 3	11 ± 1	2 ± 3	7 ± 1	9 ± 1	2 ± 1
Cholesterol (mg)	235 ± 33	146 ± 29	− 89 ± 31	236 ± 31	205 ± 35	− 31 ± 32
Fibre (g)	32 ± 3	33 ± 4	1 ± 4	30 ± 4	31 ± 3	1 ± 1
Sodium (mg/d)	2584 ± 258	2348 ± 276	− 236 ± 255	2444 ± 184	2234 ± 179	− 210 ± 156
Beverage intake						
Diet soda (ml/d)	34 ± 12	21 ± 8	− 13 ± 11	33 ± 11	17 ± 10	− 16 ± 10
Sugar-sweetened soda (ml/d)	48 ± 33	22 ± 14	− 26 ± 28	34 ± 11	51 ± 16	21 ± 14

Values are reported as the means ± SDs

Change: Absolute change from baseline to week 12

*p < *0.05*, obtained from repeated measures ANOVA

### Glucose regulation and body weight

**Fig. 3:**
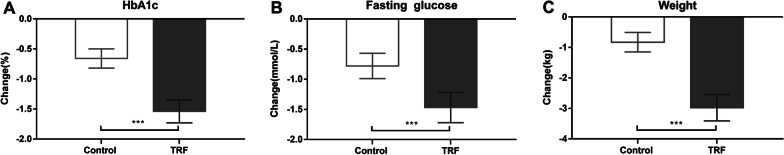
Effects of the 12-week TRF (54 participants) or control (50 participants) conditions on HbA1c, fasting glucose and weight. **A**, **B** and **C** display the data as the raw means ± SDs. ***p < 0.001*,* obtained from repeated measures ANOVA

As shown in Table 3 and Fig. 3, after 12 weeks of intervention, TRF, compared with the control, resulted in a significant reduction in HbA1c (− 1.54% ± 0.19 mmol/L [18%] vs. − 0.66% ± 0.16 mmol/L [8%]; *p* < 0.001), FPG (− 1.47 ± 0.25 mmol/L [15%] vs. − 0.78 ± 0.21 mmol/L [8%], *p* < 0.001), body weight (− 2.98 ± 0.43 kg [4%] vs. 0.83 ± 0.32 kg [1%], *p* < 0.001), BMI (− 1.64 ± 0.38 kg/m2 [6%] vs. 0.42 ± 0.24 kg/m^2^ [2%], *p* < 0.001) and HOMA-IR (− 0.51 ± 0.08 [14%] vs. − 0.12 ± 0.06 [3%], *p* = 0.02) and a significant increase in HOMA-β (0.73 ± 0.21% [24%] vs. 0.27 ± 0.10 [9%], *p* = 0.005).

**Table 3:** Glucoregulatory factors, body composition and CVD risk markers

	TRF group			Control group			*p*^a^
	Wk0	Wk12	Change	Wk0	Wk12	Change	
HbA1c (%)	8.68 ± 1.21	7.14 ± 0.89	− 1.54% ± 0.19	8.34 ± 1.09	7.68 ± 0.98	− 0.66% ± 0.16	< 0.001
FPG (mmol/L)	9.73 ± 1.38	8.26 ± 0.86	− 1.47 ± 0.25	9.54 ± 1.26	8.76 ± 1.02	− 0.78 ± 0.21	< 0.001
Insulin (mIU/L)	8.83 ± 1.19	8.40 ± 0.92	− 0.43 ± 0.18	8.84 ± 1.17	8.83 ± 0.96	− 0.01 ± 0.02	0.03
HOMA-β	3.09 ± 0.64	3.82 ± 0.65	0.73 ± 0.21	3.16 ± 0.52	3.43 ± 0.66	0.27 ± 0.10	0.005
HOMA-IR	3.71 ± 0.68	3.20 ± 0.72	− 0.51 ± 0.08	3.65 ± 0.73	3.53 ± 0.83	− 0.12 ± 0.06	0.02
Body weight (kg)	75.06 ± 4.42	72.08 ± 3.98	− 2.98 ± 0.43	74.68 ± 4.35	73.85 ± 4.26	− 0.83 ± 0.32	< 0.001
BMI (kg/m^2^)	26.42 ± 1.96	24.78 ± 1.43	− 1.64 ± 0.38	26.08 ± 2.14	25.66 ± 2.08	− 0.42 ± 0.24	< 0.001
MES total	2.12 ± 0.76	1.46 ± 0.43	− 0.66 ± 0.17	2.04 ± 0.87	1.83 ± 0.54	− 0.21 ± 0.04	0.006
MES OHA	1.72 ± 0.89	1.39 ± 0.56	− 0.33 ± 0.27	1.74 ± 0.46	1.65 ± 0.44	− 0.09 ± 0.24	0.01
MES Insulin	1.97 ± 0.62	1.65 ± 0.52	− 0.32 ± 0.26	1.91 ± 0.33	1.80 ± 0.29	− 0.11 ± 0.24	0.02
SF-12	63.56 ± 8.18	69.48 ± 7.09	5.92 ± 1.38	62.78 ± 7.49	64.49 ± 8.87	1.71 ± 1.41	< 0.001
TG (mmol/L)	2.59 ± 1.22	2.36 ± 1.18	− 0.23 ± 0.08	2.46 ± 1.18	2.33 ± 1.16	− 0.13 ± 0.06	0.03
TC (mmol/L)	5.56 ± 1.10	5.24 ± 1.05	− 0.32 ± 0.07	5.32 ± 1.10	5.17 ± 1.05	− 0.15 ± 0.06	0.01
LDL-c (mmol/L)	3.67 ± 1.24	3.25 ± 1.19	− 0.42 ± 0.13	3.57 ± 1.04	3.36 ± 0.88	− 0.21 ± 0.13	0.02
HDL-c (mmol/L)	1.24 ± 0.31	1.08 ± 0.29	− 0.16 ± 0.04	1.19 ± 0.34	1.04 ± 0.36	− 0.15 ± 0.05	0.33

Data are the means ± SDs

HbA1c, haemoglobin A1c; FPG, fasting plasma glucose; HOMA-β, homeostasis model of assessment-estimated β function; HOMA-IR, homeostasis model of assessment-estimated insulin resistance; BMI, body mass index; MES, medication effects score (MES = (actual drug dose/maximum drug dose) * drug mean adjustment factor); OHA, oral hypoglycaemic agents; TG, triglyceride; TC, total cholesterol; LDL-c; low-density lipoprotein cholesterol; HDL-c, high-density lipoprotein cholesterol

^a^Obtained from repeated measures ANOVA

Weight change directly correlated with HbA1c change between baseline and 12 weeks (r = 0.5, *p* = 0.01).

### Medication and SF-12 score

Measures of change in MES and SF-12 from baseline to the 12-week follow-up are presented in Table 3. Compared with the control, TRF resulted in a more significant reduction in the total MES (− 0.66 ± 0.17 [31%] vs. − 0.21 ± 0.04 [10%]; *p* = 0.006). The MES for oral hypoglycaemic agents in the TRF group decreased more (△ = − 0.33 ± 0.27, [19%]) than that in the control group (△ = − 0.09 ± 0.24, [5%], *p* < 0.001), which did not correlate with HbA1c (r = − 0.1; *p* = 0.34). The MES insulin change was similar in the TRF group (△ = − 0.32 ± 0.26, [16%]) and the control group (△ = − 0.11 ± 0.24, [6%], *p* < 0.001), which also did not account for any difference in HbA1c (r = − 0.2; *p* = 0.20).

The change in SF-12 score at 12 weeks was notably different (*p* < 0.001) between the TRF group (△ = 5.92 ± 1.38, [9%]) and the control group (△ = 1.71 ± 1.41 mmol/L, [3%]).

### CVD risk markers

Measures of the change in CVD risk markers from baseline to the 12-week follow-up are presented in Table 3. After 12 weeks of intervention, TRF, compared with the control, resulted in a significant reduction in TGs (− 0.23 ± 0.08 mmol/L, [9%] vs. 0.13 ± 0.06 mmol/L, [5%]; *p* = 0.03), TC (− 0.32 ± 0.07 mmol/L [6%] vs. − 0.15 ± 0.06 mmol/L [3%], *p* = 0.01) and LDL-c (− 0.42 ± 0.13 mmol/L [11%] vs. − 0.21 ± 0.13 mmol/L [6%], *p* = 0.02). However, TRF did not affect the level of HDL-c (− 0.16 ± 0.04 mmol/L vs. − 0.15 ± 0.05 mmol/L, *p* = 0.33).

### Activity

Step count remained below baseline levels at 12 weeks in the TRF group and the control group. Despite this, the total step count remained similar in both groups at 12 weeks (6139.28 ± 288.32 steps in the TRF group vs. 6272.23 ± 223.45 steps in the control group; *p* = 0.62). The activity change between baseline and 12 weeks did not correlate with weight change (r = − 0.07; *p* = 0.41).

## Discussion

This study is the first to test the effects of TRF on weight, blood sugar, and CVD risk factors in overweight patients with type 2 diabetes. Furthermore, we designed a randomized controlled trial, which provides a relatively higher level of evidence, and included type 2 diabetes patients treated only with medication as a control group and an intervention group with TRF as the treatment. Our results showed that the control group had slight improvements in related indicators due to medications, while the TRF group had more significant improvements, which was consistent with our expectations.

To our knowledge, no study has explored the effect of 10-h TRF on type 2 diabetes; thus, there are no data for comparison with our findings. Only a few trials have studied the effect of TRF on weight in obese patients. A recent 8-h TRF study reported a 2.6% weight loss after 12 weeks [24]. Similarly, 10-h TRF resulted in a 3.6% weight loss over 16 weeks [25] and a 3.0% weight decrease after 12 weeks [27]. Compared with the effects of calorie restriction with exercise in patients with glucose intolerance, the degree of weight loss observed in our trial with TRF (additional 3% reduction) was consistent. These studies found weight loss rates of 1% [32] and 3% [33]. Regarding glucose regulation, Wei tested another form of intermittent fasting (fasting mimicking diet) and reported that when the baseline fasting plasma glucose was > 5.5 mmol/L, a reduction was observed in the fasting plasma glucose [34]. However, Hutchison reported no change in fasting glucose levels among healthy overweight people after TRF [35]. Additionally, a 6-h TRF intervention in overweight prediabetic patients did not alter fasting glucose levels but did increase insulin sensitivity and β-cell function [26]. Do patients at high risk of more severe metabolic diseases benefit more from TRF than those at low risk? Our findings suggest an answer to this question. In our trial, the fasting plasma glucose level in type 2 diabetes patients was 9.73 ± 1.38 mmol/L at baseline. TRF intervention decreased fasting glucose levels by 15% and HbA1c values by 18%, approximately twice the effect of medicine, which surprised us. Moreover, notable improvements were detected in HOMA-β and HOMA-IR. However, these parameters were evaluated as short-term effects, and further research is needed to determine the long-term effects.

The degree of MES observed in our study with TRF (mean, − 31% from baseline) was greater than that in studies examining the effects of 5:2 intermittent fasting (2 nonconsecutive days/week and their usual diet for 5 days/week) with type 2 diabetes, which reported MES declines of 22% [36] and 25% [37]. Our study achieved a better result, which may be due to the presence of more severe metabolic disorders because the HbA1c baseline level was 7.3 ± 0.1% and 7.5 ± 1.4% in the two previous trials, respectively. To our surprise, compared with the MES in the control group, that in the experimental group was reduced by an additional 21%. This finding is meaningful for diabetic patients. In addition, our research showed that the TRF intervention improved the overall SF-12 score of the experimental group participants by 9%. The SF-12 [38] is a 12-item health questionnaire used to assess several areas of health-related quality of life, including physical health, mental health, and general health perceptions. This indicator has a score ranging from 0 to 100, with higher scores indicating better health. Our study indicates that the TRF intervention improved people's perception of physical function and daily activity.

The improvement in blood glucose was positively associated with changes in lipids. Therefore, we hypothesised that the level of dyslipidaemia would gradually recover with the reduction in blood sugar levels. In our study, none of the participants used statins or fibrates. Total cholesterol, triglycerides and LDL cholesterol in the control group decreased by 5%, 3% and 6%, respectively. However, the reduction in the TRF group was almost twice that in the control group. There were obvious reductions in triglycerides (9%), total cholesterol (6%), and LDL cholesterol (11%) in the TRF group of this study. The effect of intermittent fasting on blood lipids varies widely. Most studies have shown no effect on blood lipid indicators [24, 39]. HDL cholesterol is also not affected by these diets, although one study observed a slight increase [40]. Neither 4-h TRF nor 6-TRF intervention affected blood lipid levels in obese adults at week 8 [28]. However, it is important to note that the participants in these reports did not have hyperlipidaemia. In a recent study exploring 10-h TRF in patients with metabolic syndrome, the single-arm trial revealed decreases in total cholesterol (7%) and LDL-C (11%) compared with baseline. The results in our experiment were comparable to those of this trial.

Many people are concerned with adverse effects. One crossover 6-h TRF study included reports of detected vomiting (n = 1), headache, diarrhoea, and increased thirst (n = 2) [26]. Another study on 8-h TRF reported a nonsignificant increase in the incidence of adverse events, such as nausea, diarrhoea and dizziness [41]. Additionally, 10-h TRF in individuals with metabolic syndrome was associated with muscle discomfort (n = 1), which was considered unrelated to the experiment [27]. Participants in our trial of 10-h TRF intervention did not experience any of these adverse events. The number of hypoglycaemic events was zero in the TRF group. In our study, adherence to the TRF intervention was very good.

There are several hypotheses about the mechanism of TRF-induced metabolic benefits. One study observed that restriction of feeding could prevent weight gain, dyslipidaemia and fatty liver disease by reversing the phase of clock genes in peripheral organs in mice [42]. However, other studies found that natural eating patterns only weakly affect the body clock. Instead, in normally fed mice, the central pacemaker in the brain may phase the peripheral organs through pathways unrelated to feeding behaviour. Results in rats and mice showed that food rhythm is not necessary to support in sync peripheral organs [43], and in the absence of food rhythm, adrenaline connects the central and peripheral clock signal [44]. The results of human studies have also yielded no positive results. A crossover study compared the effects of early (8 am to 5 pm) and late (12 pm to 9 pm) time-restricted eating on glucose tolerance. The authors demonstrated that time-limited feeding improved glycaemic responses regardless of mealtime (late or early) [35]. In a study by Gill S [25], no additional effect of daily feeding time was observed, but the benefits of TRF were found to be due to energy restriction, which is consistent with our study. Our results showed that 10 h of daily eating reduced caloric intake without deliberate caloric counting. As a result, the participants in the TRF group lost approximately 4% of their bodyweight and showed improvements in other indicators, which was consistent with Gabel K [24] and Cienfuegos S [28]. If TRF can inadvertently lead to reduced calorie intake under normal conditions, it is a relatively attractive way to reduce calorie intake because individuals and doctors do not need to employ expensive and laborious methods to accurately track calories. Therefore, TRF is an effective way to improve health.

## Limitations

This study has several limitations. First, although this was a study with a relatively large sample, the sample size could be further expanded. Second, the time of intervention was short, and further follow-up is needed to observe the long-term results of TRF. Third, a subgroup design was not implemented for the legacy effect of TRF in our study. Fourth, the BMIs of the patients in this study were relatively low, and racial restrictions may have limited wider global use. Fifth, self-reports, such as food records and adherence to the intervention, were included. Last, we did not design a crossover study. A crossover trial has the advantages of self-matching, such as reducing the impact of individual differences on processing factors.

## Conclusion

Our research is the first randomised controlled trial to explore the effects of TRF in humans with type 2 diabetes. Our study showed that 10-h TRF reduced body weight and blood glucose and improved insulin sensitivity in overweight patients with type 2 diabetes. These results occurred without deliberate attempts to increase physical activity and change the quality or quantity of diet. Importantly, we also found significant improvement in CVD risk markers (triglycerides, total cholesterol and LDL cholesterol) without the use of stains or fibrates. Additionally, when all of the above indicators were significantly controlled, after the TRF intervention, the dosage of hypoglycaemic drugs in the experimental group of participants was significantly reduced, and their perception of physical functions and daily activities were improved. Furthermore, the good compliance, high level of adherence to TRF, and low dropout rate in our study indicate that the 10-h window for TRF may be feasible for patients with type 2 diabetes to follow.

## Supplementary Information

**Additional file 1: Figure S1**. The number of adherent days to the dietary regimen for the TRF group

## Data Availability

The datasets used during the present study are available from the corresponding author upon reasonable request.

## Abbreviations

BMI: Body mass index
FPG: Fasting plasma glucose
HbA1c: Haemoglobin A1c
HOMA-β: Homeostasis model of assessment-estimated β function
HOMA-IR: Homeostasis model of assessment-estimated insulin resistance
HDL: High-density lipoprotein
LDL: Low-density lipoprotein
MES: Medication effects score
OHA: Oral hypoglycaemic agents
TG: Triglyceride
TC: Total cholesterol
