# Supplementary figures and images for: Time-restricted feeding improves blood glucose and insulin sensitivity in overweight patients with type 2 diabetes: a randomised controlled trial
Source: Nutr Metab (Lond). 2021 Oct 7;18:88. doi: 10.1186/s12986-021-00613-9 (PMC8499480; doi:10.1186/s12986-021-00613-9)

Figure S1.The number of adherent days of the dietary regimen for the TRF group.

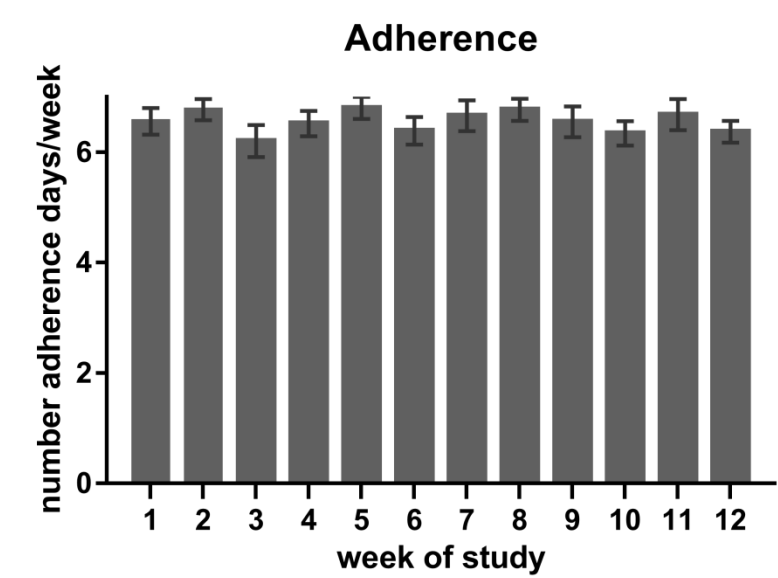

Supplement: Supplementary file 1 — Additional file 1: Figure S1. The number of adherent days to the dietary regimen for the TRF group [file 12986_2021_613_MOESM1_ESM.pdf]
